# Lateral Impact Performance of Cold-Formed Steel L-Shaped Built-Up Columns

**DOI:** 10.3390/ma18194548

**Published:** 2025-09-30

**Authors:** Mengyao Li, Jinshan Sun, Yi Hu, Liqiang Jiang, Shizhong Zhou, Guangwei Dai, Ning Wu

**Affiliations:** 1 Hubei Key Laboratory of Blasting Engineering, Jianghan University, Wuhan 430056, China; 20241200668@csuft.edu.cn (M.L.); jianglq2019@csu.edu.cn (L.J.); 2 School of Civil Engineering, Central South University of Forestry and Technology, Changsha 410004, China; hyi_1991@163.com; 3 School of Civil Engineering, Central South University, Changsha 410075, China; 15034738691@163.com; 4 China Construction Third Engineering Bureau Urban Construction Co., Ltd., Changchun 130000, China; 13662120170@163.com (G.D.); 13596042389@163.com (N.W.)

**Keywords:** CFS, L-shaped built-up column, impact performance, numerical simulation, parametric analysis

## Abstract

Blasts, vehicle collisions, and other unexpected incidents may cause lateral impacts on building structures, which threaten their safety. This paper investigates the impact resistance of cold-formed steel (CFS) L-shaped built-up columns (LBC). Firstly, a finite element model (FEM) was established and validated through experiments conducted by the authors. Then, a parametric analysis was conducted to quantify the effects of axial compression ratio, impact velocity, and dimensions on the impact response. The results indicated that: (1) The peak lateral impact force of the specimens presented a significant nonlinear trend with increasing axial compression ratio, and an optimal axial compression ratio was found as about 0.3. (2) Higher impact velocity intensified both force and displacement responses of the specimens, and both lateral impact peak force and maximum displacement increased significantly with the impact velocity. When the impact velocity rose from 3.13 m/s to 6.26 m/s, the peak force and maximum displacement increased by an average of 38.2% and 96.5%, respectively. (3) Increasing the cross-sectional dimensions and steel thickness, and reducing screw spacing, could significantly enhance the impact resistance and deformation capacity of the specimens. This study reveals the failure mechanism of such members and the laws of parameter influence, which can be used for impact design of CFS-LBC.

## 1. Introduction

While modern building structures are pursuing functional diversity and space utilization efficiency, their safety is also facing increasingly complex challenges. Among these, sudden lateral impact forces, such as vehicle collisions, explosive shock waves, or falling heavy objects, are one of the key threats that can cause severe structural damage or even collapse [1,2,3,4,5,6]. Such forces, characterized by short duration and high energy, pose a severe test to structural components, especially columns located at critical positions or with special shapes [7,8,9,10]. Particularly in irregular spaces such as building corners, traditional rectangular columns often protrude from the wall, which affects the usability and esthetics [11,12,13]. Cold-formed steel (CFS) has become an important choice for realizing green and low-carbon buildings owing to its significant advantages, such as light weight, high strength, flexible cross-sectional forms, and ease of industrial production and assembly construction [14,15,16,17,18,19]. The emergence of L-shaped cross-section special-shaped columns has cleverly solved the problem of column arrangement in corner spaces, achieving the effect of “columns not protruding from walls” and greatly improving the regularity and utilization rate of space [20,21,22,23]. Forming L-shaped cross-section columns by splicing CFS is undoubtedly an ideal solution that combines the green advantages of materials and space adaptability [11]. Nevertheless, there is still a lack of systematic and in-depth research on the dynamic response, failure mechanism, and key influencing factors of such built-up columns when they are subjected to accidental lateral impacts under axial compression service conditions. The quantitative evaluation of their impact resistance and optimized design remains a critical scientific issues that need to be addressed urgently, which is directly related to the safety of such structures in practical applications.

In recent years, researchers have carried out comprehensive explorations on the performance of structural components under impact forces [24,25,26,27,28,29]. Chen et al. [30] performed drop hammer tests and established an experimentally calibrated finite element model (FEM) to investigate the dynamic response and damage evolution of recycled aggregate concrete-filled steel tube columns under lateral impact. They found that the peak mid-span deflection lagged behind the peak impact force and increased parabolically with the impact velocity. The replacement ratio of recycled aggregate had a limited influence on the overall response, while the boundary conditions and the steel ratio of the cross-section significantly affected the plateau value of the impact force. Furthermore, they proposed a rapid damage assessment curve based on the elastoplastic rotation angle. Huang et al. [31] conducted pendulum impact tests combined with LS-DYNA simulations to compare the impact resistance of geopolymer concrete columns reinforced with basalt fiber rebars and ordinary concrete columns reinforced with steel bars. They pointed out that under low-velocity impact, steel bar columns suffered less damage due to their high stiffness; under high-velocity impact, fiber rebar columns performed better, taking advantage of their high-strength elasticity. Additionally, geopolymer concrete could be used as an equivalent substitute for ordinary concrete. Guo et al. [32] designed reinforced recycled aggregate concrete simply supported beams with different replacement ratios of recycled coarse aggregate, and carried out static loading and drop hammer impact tests to study their dynamic characteristics and energy dissipation under impact. The findings showed that under static loading, the beams exhibited typical flexural failure, and the static resistance and stiffness decreased slightly with the increase in the replacement ratio. Under impact loading, the failure was controlled by bending. The mid-span displacement increased with the increase in the replacement ratio, while the global damage energy dissipation and its ratio decreased slightly with the increase in the replacement ratio. Wang et al. [33] conducted lateral impact tests on 22 circular concrete-filled steel tube members and established a FEM to study their performance under lateral impact, taking into account parameters such as axial load level, confinement factor, and impact energy. The results indicated that specimens with a larger confinement factor exhibited ductile failure, while those with a smaller confinement factor showed brittle failure. This phenomenon is mainly attributed to the confinement effect of the steel tube, a fundamental mechanism in steel–concrete composite structures. The confinement provided by the steel tube significantly enhances the compressive strength and ductility of the concrete by retarding its lateral expansion and inducing a favorable triaxial stress state [34,35]. Both the confinement factor and axial load level had an influence on the critical fracture energy, and the established FEM could well predict the impact behavior of the members.

In the field of CFS, research has mostly focused on the axial or lateral impact responses of beams and columns with conventional cross-sections, revealing their failure modes, energy absorption characteristics, and the influences of some design parameters [36,37,38,39]. Bambach et al. [40] studied the failure mechanism and energy dissipation performance of hollow and concrete-filled square steel tubes under low-velocity transverse impact through drop hammer tests and an elastoplastic theoretical model. The findings demonstrated that concrete filling could restrain local deformation and significantly improve the flexural bearing capacity of the cross-section, but for non-compact cross-sections, it would cause tearing in advance, thereby reducing ductility and energy absorption. The model could well predict deformation and bearing capacity. Al-Thairy et al. [41] studied the response and failure modes of axially compressed steel columns under transverse impact by establishing and validating an ABAQUS model. The results indicated that the failure was mainly dominated by overall buckling, and local deformation was only a consequence of it. The failure of the column mainly depended on the magnitude of the kinetic energy of impact, while the combined effect of impact mass and velocity was relatively small. Liang et al. [14] conducted a comparative analysis of the response differences between cold-rolled dimpled square steel tubes and flat-walled square steel tubes under low-velocity transverse impact using an explicit FEM. The findings demonstrated that the average impact force of the dimpled columns increased by 32.5%, the crushing efficiency improved by 24.4%, and their stability under axial compression was better. The dimple geometry mainly reduced the peak force, while material hardening enhanced the overall strength. For special-shaped columns, especially reinforced concrete special-shaped columns, there is abundant research on their seismic performance, but studies on their behavior under impact forces are relatively scarce [42,43,44,45,46]. Notably, research on the mechanical behavior of complex cross-section columns formed by splicing thin-walled steel sections under the combined action of axial compression and lateral impact is even rarer.

Existing studies either focus on a single load condition or fail to fully consider the splicing interface effect, cavity structure, and the sensitivity of key parameters (such as impact point height and initial axial compression level) [47,48,49]. These studies have laid a foundation for our understanding of the impact dynamics of thin-walled steel structures, but they also clearly indicate that there is insufficient research on the performance of CFS L-shaped built-up columns (LBCs), which offer both spatial efficiency and material advantages, under complex stress paths. In particular, the quantitative influence laws of key design parameters such as axial compression ratio, impact velocity, and steel thickness on their impact resistance have not been fully elucidated.

Therefore, this paper focuses on the key issue of the performance of CFS-LBC under lateral impact forces. It mainly adopts the ABAQUS explicit dynamics module to establish a high-precision three-dimensional FEM, and ensures the reliability of calculations through detailed model construction and verification. On this basis, a systematic parametric analysis is carried out, with the core focus on: (1) the influence of axial compression ratio on impact response and failure modes; (2) the performance differences caused by changes in lateral impact velocity; (3) the regulatory effect of steel thickness, screw spacing and cross-section size on the impact resistance of components. This study aims to thoroughly analyze the influence of laws of the above key parameters on the impact force time history, displacement development, and final failure mode of built-up columns, as well as to reveal their dynamic response characteristics and failure mechanisms. This study further seeks to provide a scientific basis and design reference for evaluating and improving the safety performance of such structural components—those with both green environmental protection and space adaptability advantages—under extreme accidental loads.

## 2. FEM Verification

**Figure 1:**
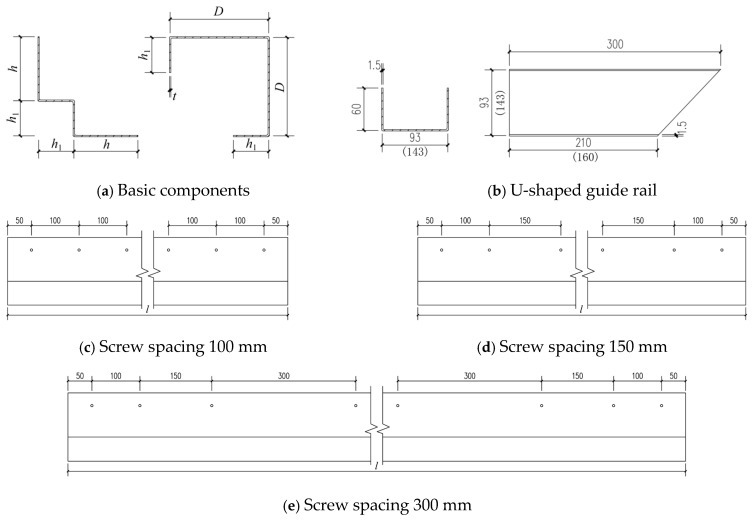
Details of built-up column dimensions.

The specimens used in this study were the M-series medium-length columns previously developed by our research group [11]. The self-tapping screws are of ST4.8 grade with a designed length of 19 mm. All components were constructed strictly in accordance with the measured dimensions, and the outer radius of all corner folds was uniformly set to 2 mm. Specific dimensional details are shown in Figure 1 and Table 1. The specimen numbering rule is as follows: “M-140-100-1.2”, where “140” represents the web height, “100” indicates the screw spacing, and “1.2” denotes the steel thickness, with all units in mm. Testing was conducted on a 200 T electro-hydraulic servo-control testing machine. The specimens were connected to U-shaped tracks at both ends via self-drilling screws, simulating a semi-rigid boundary condition. Axial displacement-controlled loading was applied. The validation herein, while utilizing the same test basis, is fundamentally aimed at enabling a novel investigation into the coupled effects of axial compression and lateral impact, a critical scenario not addressed in prior work.

Based on the previous axial compression test results within the author’s research group, this chapter verified the accuracy of the FEM by comparing simulation results with test data and laid a foundation for subsequent impact simulations [11].

### 2.1. Comparison of Failure Phenomena

**Figure 2:**
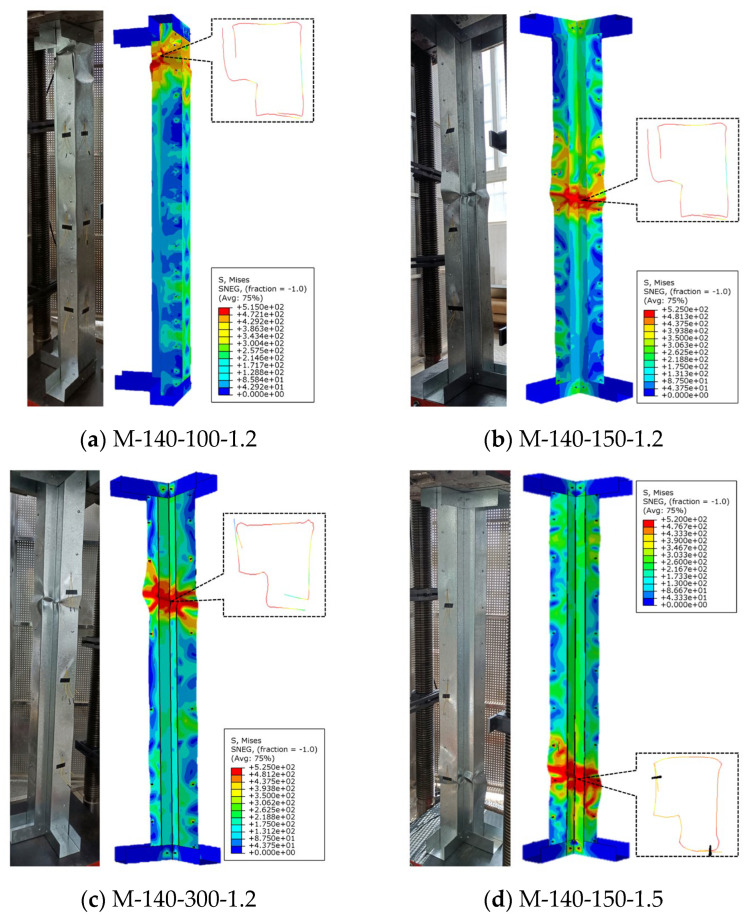
Comparison of specimen failure modes.

The failure characteristics of the specimens from the finite element analysis were compared with the experimental results, as illustrated in Figure 2. It is evident from the figure that the specimens displayed a failure mode combining local and distortional buckling, with no torsional buckling observed in any specimen. Moreover, the failure positions of the specimens were relatively consistent with those in the experiments. During the experiments, the two basic components showed significant separation at the failure position of the specimens, and this phenomenon was accurately simulated by the FEM. In addition, none of the screws in any of the specimens were pulled out or fractured during the finite element analysis, which is consistent with the experimental observations.

### 2.2. Comparison of Load–Displacement Curves

**Figure 3:**
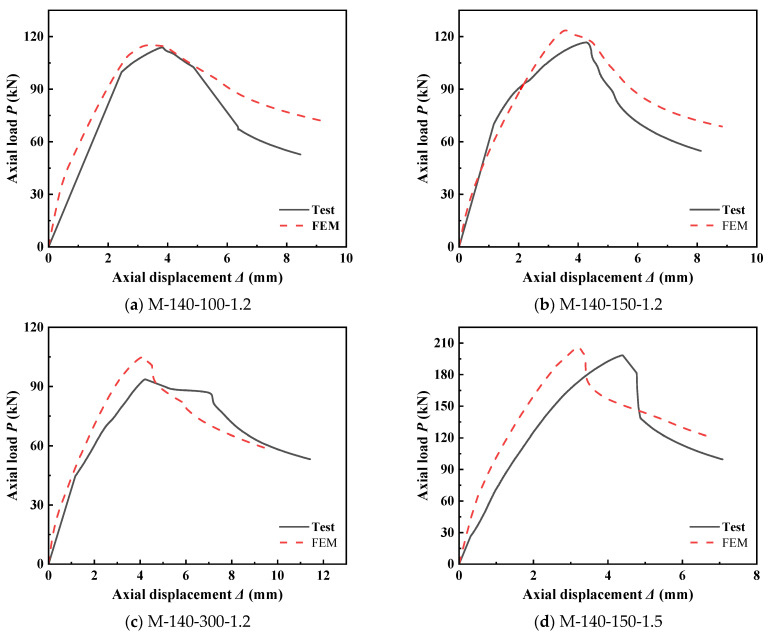
Comparison of axial load–displacement curves of specimens.

The load-axial displacement curves of the specimens from the finite element analysis were compared with those from the experiments, as depicted in Figure 3. It is evident from the figure that in the early stage of loading, the slope of the experimental load-axial displacement curve was relatively small, and then it returned to a normal slope, while the finite element analysis curve showed a linear growth trend throughout. The main reason is that during the assembly of the experimental specimens, certain inevitable gaps were formed when the ends of the built-up components were connected to the guide rails using self-tapping screws. Thus, in the early loading stage, these gaps underwent a compaction process, leading to uneven stress on the specimens and consequently a smaller curve slope. However, the finite element analysis was conducted under relatively ideal conditions, so no similar phenomenon occurred. In addition, in the middle stage of loading, the slope of the finite element analysis curve was slightly larger than that obtained from the experiment, indicating that the overall stiffness simulated by the FEM was greater than that of the experimental specimens. In the later stage of loading, the slope of the experimental curve decreased more rapidly compared to that of the finite element analysis, which suggests that the stiffness degradation of the specimens during the experiment was more significant than that in the finite element analysis model. Overall, the load–axial displacement curve from the finite element analysis is in good agreement with that from the experiment.

### 2.3. Comparison of Ultimate Capacity

As can be seen from Table 2, due to factors such as splicing errors, centering errors of the test specimens, and initial material defects, the simulated ultimate capacity values of the specimens are all greater than the actual test loading values. The error between the finite element results and the test results of the specimens is within 10%. Considering the actual errors in the test and the operational errors of the specimens, the error between the specimen simulation and the actual test is acceptable overall, which proves the accuracy and feasibility of the finite element modeling method adopted in this study.

## 3. Establishment of FEM

Based on the axial compression model verified in Chapter 2, this chapter established an FEM for simulating lateral impact by introducing a drop hammer component and modifying the analysis steps to couple axial compression with impact forces.

### 3.1. Creation and Assembly of Model Components

All components required for the model were created in the Part module of ABAQUS/CAE. The built-up steel components and U-shaped guide rails were simulated using S4R elements (4-node linear shell elements with reduced integration), while self-tapping screws and the drop hammer were modeled with C3D8R elements (8-node linear hexahedral elements with reduced integration). The specific dimensions of the specimens are shown in Figure 1 and Table 1. The three-dimensional models were generated using the extrusion command, and screw holes and reserved holes were precisely created at corresponding positions on the steel components via the hollow extrusion command for simulation. In addition, a cylindrical component representing the impact head of the drop hammer was specifically designed. This component was defined as a rigid body with a diameter of 100 mm and a constant mass of 339 kg [50], which is used to apply lateral impact forces.

**Figure 4:**
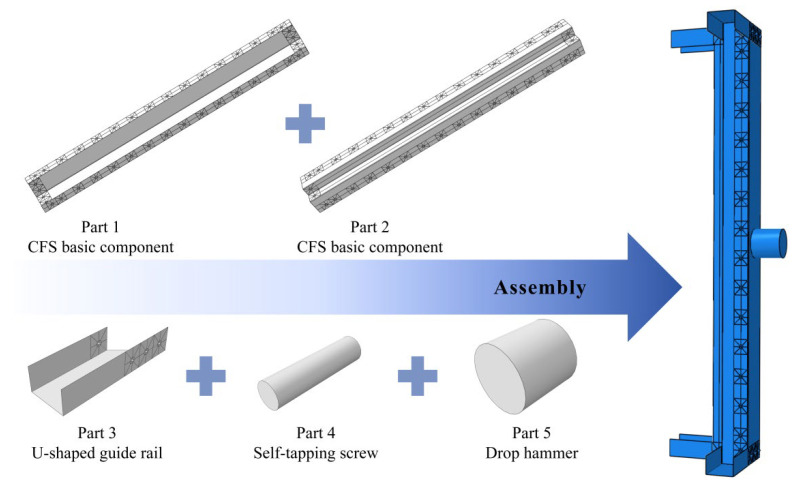
Assembly of the built-up column.

In the Assembly module, all components were spatially positioned and assembled. First, the translation, rotation, and array commands were used to accurately assemble the steel components, U-shaped guide rails, and self-tapping screws into a complete main structure of the specimen in strict accordance with their actual structural relationships. Subsequently, the drop hammer cylinder was assembled and positioned such that the center point of its end face was precisely at the mid-span height of the column, with a 1 mm gap maintained from the web surface of the specimen, and the impact direction was perpendicular to the web surface. To address the local geometric discontinuities and potential stress concentrations caused by the openings, mesh refinement was performed in the areas around the holes to ensure mesh quality and calculation accuracy, particularly in regions of stress concentration. The complete solid model after assembly, including all structural components and the positioned drop hammer, is illustrated in Figure 4.

### 3.2. Model Material Properties

**Figure 5:**
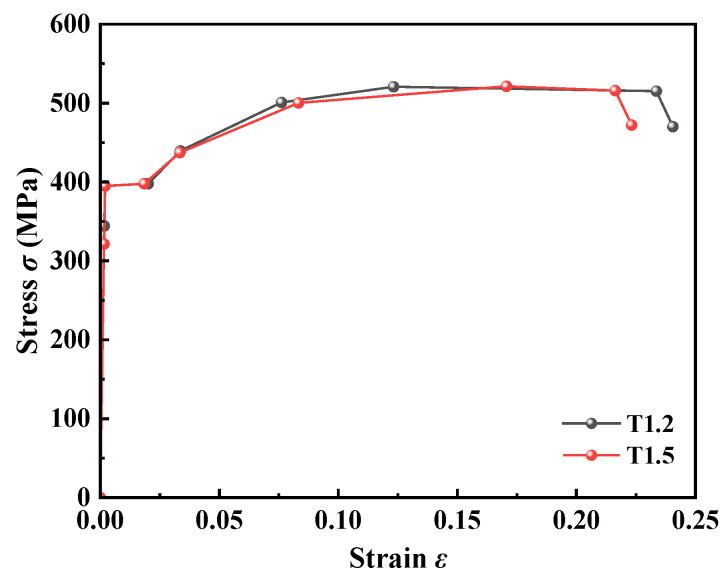
Stress–strain relationship curve.

The material parameters of the FEM were input entirely in accordance with the experimentally measured results, and the material properties of the specimens were derived from previous tests conducted by the research group [11]. Tensile tests showed that for 1.2 mm thick steel, the average yield strength and Young’s modulus were 395.1 MPa and 2.01 × 10^5^ MPa, respectively; for 1.5 mm thick steel, the yield strength and Young’s modulus were 395 MPa and 2.03 × 10^5^ MPa, respectively, with a Poisson’s ratio of ν = 0.3. The stress–strain relationship of the specific material constitutive model is given in Table 3, and the stress–strain curve is illustrated in Figure 5. Based on the results of axial compression tests, no shear or pull-out failure of the screws was observed, so the self-tapping screws were simplified as a linear elastic material (with a yield stress of 400 MPa and a plastic strain of 0). The self-tapping screws material parameters were set as follows: density 7.8 g/cm^3^ and Poisson’s ratio 0.3.

### 3.3. Constraints and Loads

**Figure 6:**
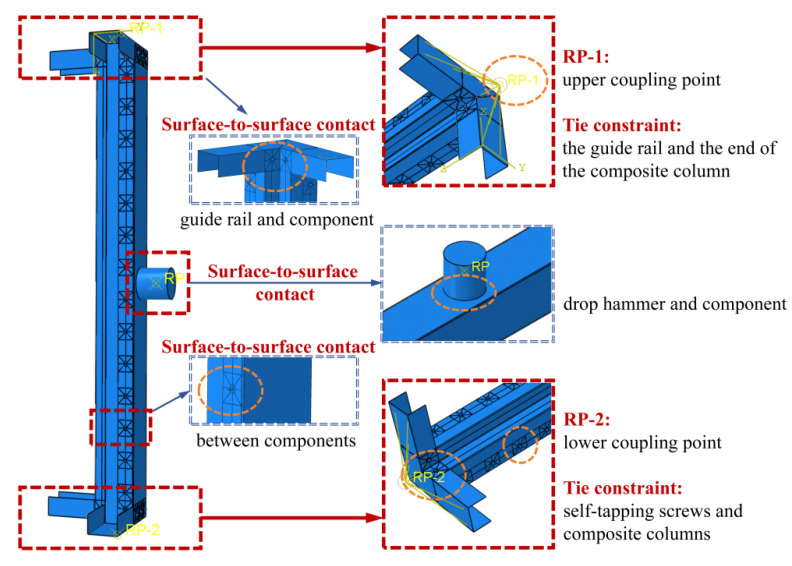
Interaction between components.

In the Interaction module, the mechanical interactions between various components were defined. Surface-surface contact was established between the basic steel members as well as between the members and the U-shaped guide rails to prevent mutual penetration of the plates; the tangential behavior of the contact surfaces was set to frictionless, and the normal behavior adopted the “hard” contact criterion. Rigid connections were achieved through tie constraints between the guide rails and the ends of the built-up columns, as well as between the self-tapping screws and the pre-reserved holes in the built-up columns. Meanwhile, to simulate the drop hammer impact, surface-surface contact was established between the drop hammer and the web surface at the mid-span of the column, with a tangential friction coefficient of 0.2, and the “hard” contact criterion was still used for the normal direction. The interactions between components are shown in Figure 6.

**Figure 7:**
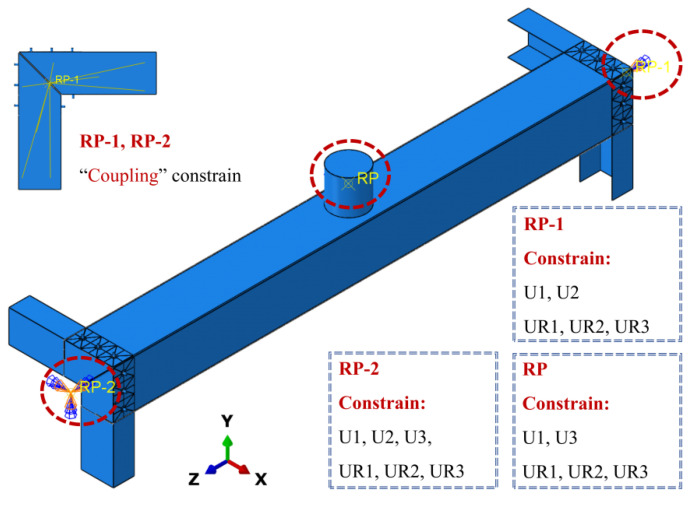
Boundary conditions.

In the Load module, boundary conditions and loading mechanisms were set. According to the characteristics of the test device, reference points RP-1 (lower end) and RP-2 (upper end) were established at the centroids of the upper and lower end faces of the model, respectively. The RP-1 and RP-2 were connected to the outer surfaces of the corresponding end guide rails through coupling constraints, and the inner surfaces of the guide rails were tied to the ends of the built-up columns. A fixed constraint was applied to the lower reference point RP-1 (constraining all six degrees of freedom), while the upper reference point RP-2 was constrained in five degrees of freedom except for the axial displacement U3, so as to simulate the axial compression loading boundary. The drop hammer reference point RP was constrained in five degrees of freedom, except for the impact direction U2. A multi-step analysis setup was adopted in the analysis process: first, an initial static general analysis step was established, and an axial pressure was applied to RP-2 to achieve the target axial compression ratio; subsequently, a dynamic explicit analysis step was established to simulate the impact process. To realize data transfer between the two analysis steps, the restart output was set in the static analysis step, and field variables such as stress and deformation in the preloaded state were saved by writing a restart file. At the start of the explicit analysis step, the initial stress state of the structure was inherited through the restart read-in function; meanwhile, the axial pressure on RP-2 was kept constant, and an initial velocity in the negative U2 direction was assigned to the drop hammer reference point (RP) to simulate lateral impact. Details are shown in Figure 7.

### 3.4. Mesh Division

**Figure 8:**
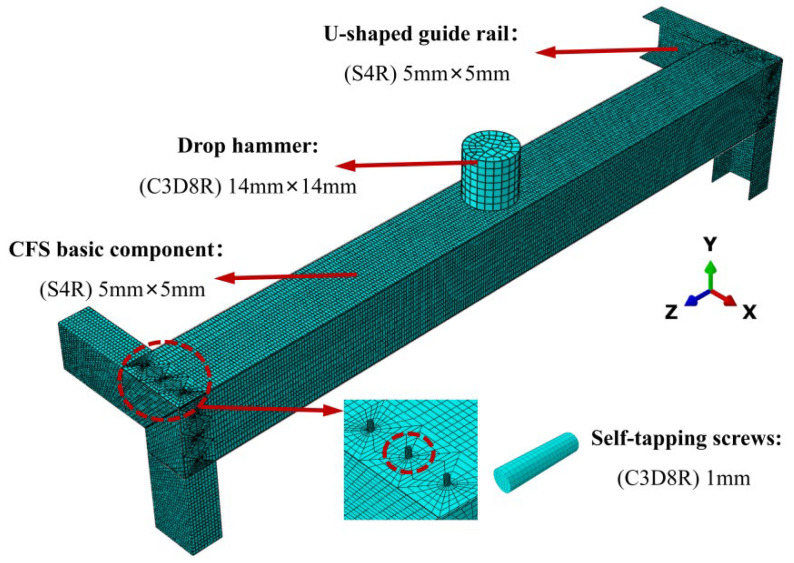
Component meshing.

In this study, a structurally optimized mesh division method was adopted to ensure calculation accuracy and convergence. The ABAQUS FEM is sensitive to mesh quality; improper division can significantly affect the accuracy of results and even cause calculation interruptions. Due to the presence of numerous open-hole regions in the built-up column components, low-quality mesh elements are prone to forming around the holes, requiring targeted local refinement. The specific mesh scheme is as follows: the basic steel components and U-shaped guide rails use a uniform mesh density of 5 mm × 5 mm (length × width); due to their geometric complexity, the self-tapping screws are meshed with a refined size of 1 mm × 1 mm; the drop hammer impact head, as a rigid component, has its surface mesh size set to 14 mm × 14 mm. The mesh division morphology of each component is detailed in Figure 8. This scheme effectively balances the need to capture stress concentrations in open-hole regions and the overall computational efficiency.

## 4. Parameter Analysis

A parametric study was conducted in this chapter to systematically evaluate the influence of laws of axial compression ratio, impact velocity, and specimen characteristics on the impact response of LBC. A total of 36 sets of numerical models were established, covering axial compression ratios (*n* = 0.1, 0.3, 0.6), impact velocities (*v* = 3.13 m/s, 6.26 m/s), and six types of specimen dimension combinations (M-140-100-1.2, M-140-150-1.2, M-140-150-1.5, M-190-100-1.2, M-190-150-1.2, and M-190-150-1.5). By analyzing the characteristics of specimen failure modes, the influence of axial compression ratio, velocity sensitivity, and the influence of specimen dimension parameters, an impact-resistant design method for the new-type cross-section was finally proposed.

### 4.1. Specimen Failure Characteristics

**Figure 9:**
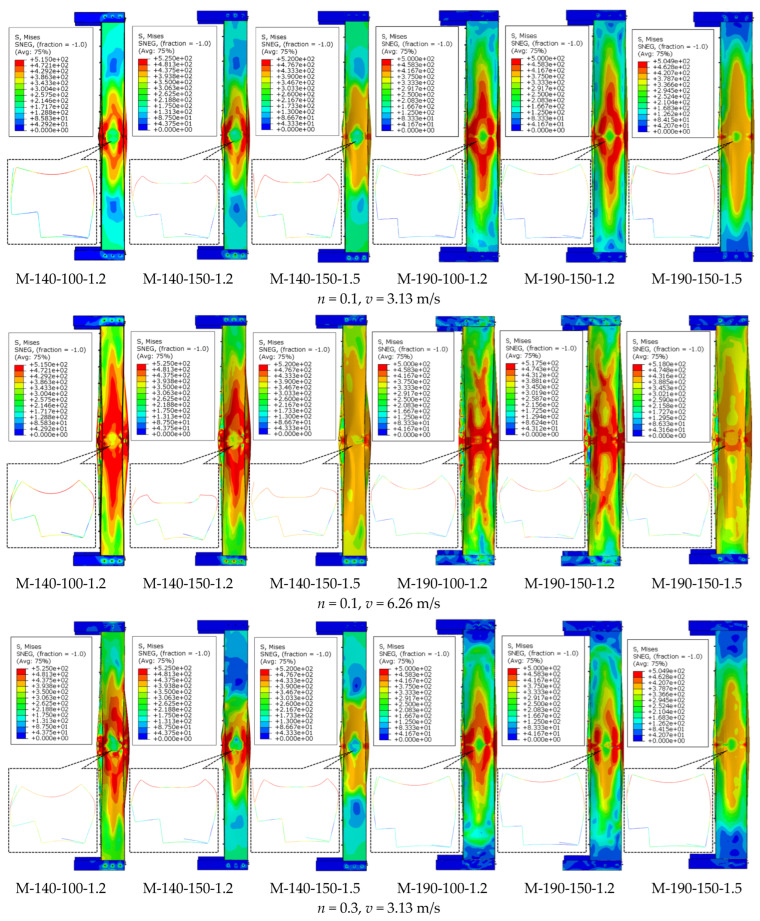

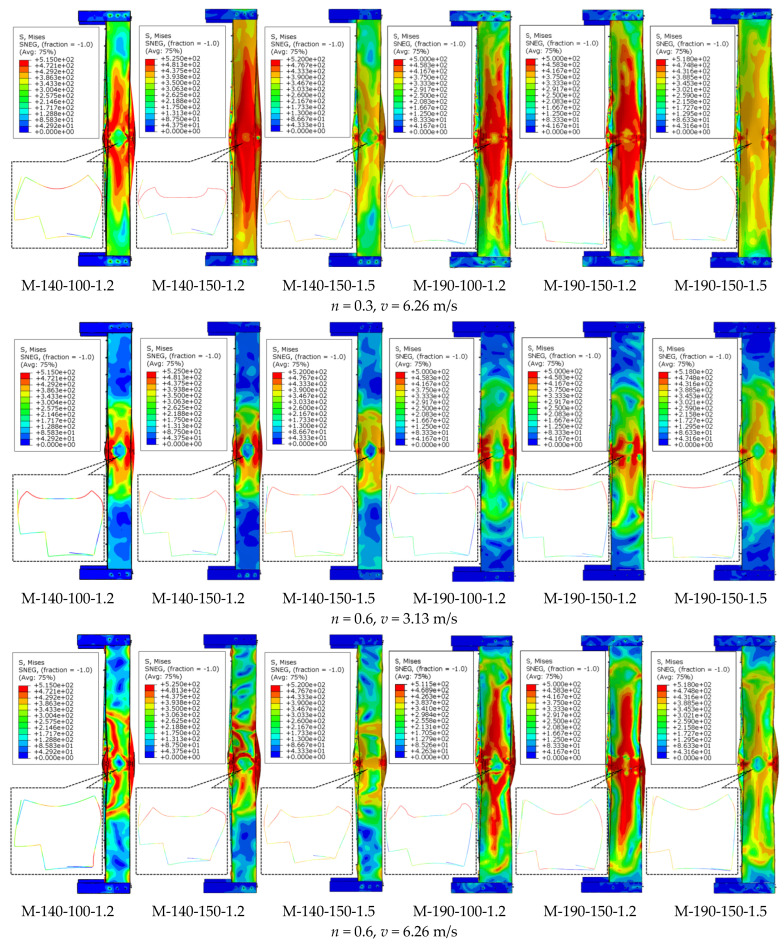
The failure characteristics of the specimen.

Through the systematic analysis of stress nephograms of 36 sets of specimens, the typical failure characteristics of CFS-LBC under impact forces can be observed. As clearly shown in Figure 9, the maximum stress concentration areas of all specimens occur at the impact contact points and the bending positions of the L-shaped cross-sections, where plastic deformation first occurs and obvious stress concentration zones are formed. With the axial compression ratio rising from 0.1 to 0.6, the stress concentration areas expand significantly, and the number of high-stress areas increases, indicating that the stress state of the members tends to be complex. Especially under the condition of high axial compression ratio, the overall stress level of the specimens increases significantly, and the cooperative working capacity of the cross-sections decreases to some extent. The increase in impact velocity also affects the failure characteristics. At an impact velocity of 6.26 m/s, the stress concentration areas are larger in scope and have higher stress values, indicating that more materials enter the plastic state and energy dissipation is more sufficient. Specimens with different cross-sectional dimensions exhibit distinct stress distribution characteristics. The M-190 series specimens show relatively uniform stress distribution, while the M-140 series specimens display more obvious stress concentration. Specimens with a larger steel thickness demonstrate a stronger local load-bearing capacity, characterized by a relatively concentrated stress distribution. The screw spacing parameter also affects the stress transfer effect. Specimens with smaller spacing exhibit a more continuous stress transfer path, and the two basic components show better cooperative working performance; in contrast, specimens with larger spacing present obvious stress concentration at the connection joints. Comprehensively, the failure process of the specimens exhibits a regular characteristic, developing from local yielding to overall plasticity. The areas near the impact points and at the cross-sectional bending positions are always the weakest links, and the stress development in these areas determines the final failure mode of the specimens.

### 4.2. Effect of Axial Compression Ratio

The axial compression ratio is one of the key factors affecting the impact resistance of CFS-LBC. In this section, the mechanism of the axial compression ratio was systematically explored by analyzing the variation laws of the peak impact force, mid-span lateral displacement, and axial displacement of specimens under different axial compression ratios. Table 4 presents the axial loads applied for different axial compression ratios corresponding to the six types of specimens.

**Figure 10:**
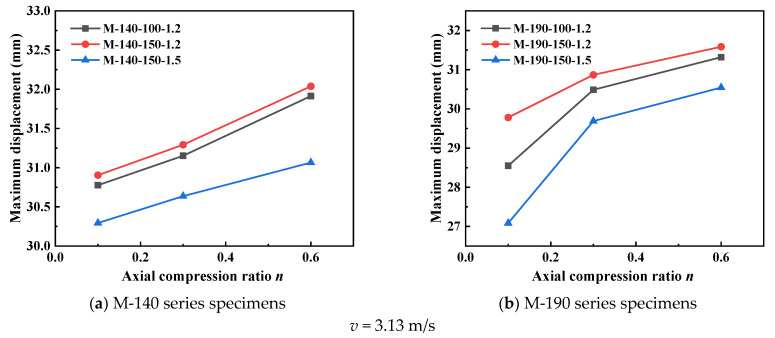

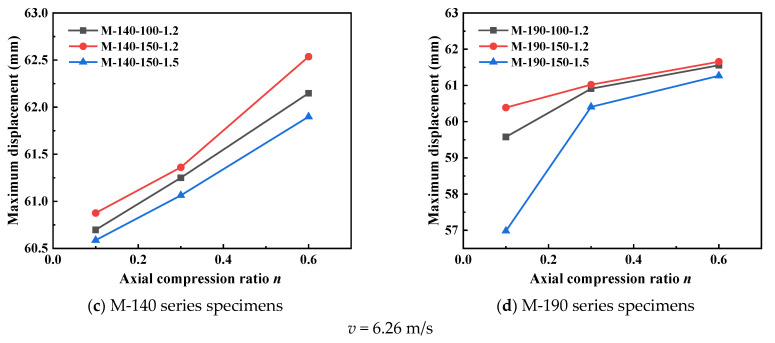
Mid-span lateral displacement of specimens with different axial compression ratios.

The data on maximum mid-span lateral displacement in Figure 10 shows that with an increase in axial compression ratio, the lateral displacement of all specimens presents a monotonically increasing trend. At an impact velocity of 3.13 m/s, the lateral displacement of the M-190-100-1.2 specimen increases from 28.549 mm (at an axial compression ratio of 0.1) to 31.32 mm (at an axial compression ratio of 0.6), with an increase rate of 9.7%. At an impact velocity of 6.26 m/s, the displacement increase is more significant; for example, the displacement of the M-190-150-1.5 specimen increases from 56.982 mm to 61.266 mm, with an increase rate of 7.5%. This phenomenon indicates that the increase in axial compression ratio intensifies the P-Δ effect, leading to a substantial increase in the second-order bending moment, thereby reducing the stiffness and stability of the members. Meanwhile, the displacement response is more sensitive under high-speed impact conditions, which indicates that there is a coupled amplification effect between impact energy and axial compression ratio.

**Figure 11:**
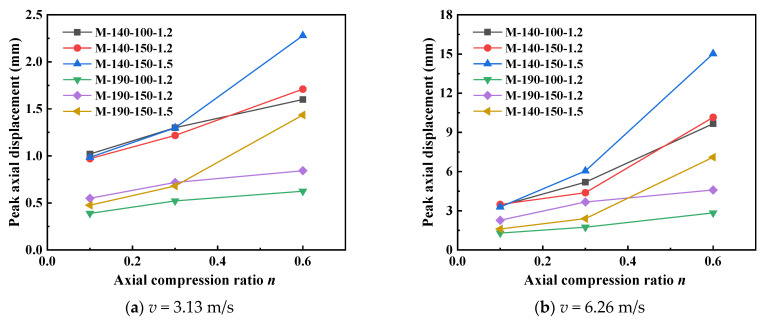
Peak axial displacement of specimens with different axial compression ratios.

The axial displacement data in Figure 11 further reveals the influence of the axial compression ratio on the compressive deformation of the members. Under an impact velocity of 3.13 m/s, the axial displacement of the M-140-150-1.5 specimen increases from 0.985 mm (at an axial compression ratio of 0.1) to 2.279 mm (at an axial compression ratio of 0.6), with an increase rate as high as 131.4%. Under the high-speed impact of 6.26 m/s, the axial deformation is more drastic: for the same specimen, the axial displacement increases from 3.293 mm to 15.026 mm, showing an extremely high increase rate of 356%. This indicates that a high axial compression ratio significantly increases the axial shortening deformation of the members, accelerates the material’s transition into the plastic stage, and reduces the energy dissipation capacity of the members. The sharp increase in axial displacement also reflects the degradation of the overall stability of the members, which mutually confirms the variation law of lateral displacement.

**Figure 12:**
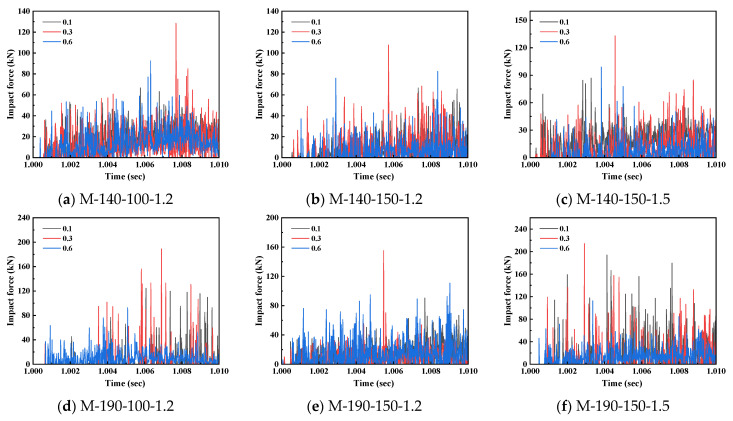
Impact force-time history curves of specimens with different axial compression ratios.

From the lateral impact force-time history curves in Figure 12, it can be observed that at an impact velocity of 3.13 m/s, the peak impact force of all specimens exhibits a nonlinear variation trend of first increasing and then decreasing with the increase in axial compression ratio. Taking the M-190-150-1.5 specimen in Figure 12f for instance: when the axial compression ratio increases from 0.1 to 0.3, the peak force rises from 193.92 kN to 214.27 kN, with an increase rate of 10.5%; nevertheless, when the axial compression ratio further ascends to 0.6, the peak force drops sharply to 112.68 kN, showing a decrease rate as high as 47.4%. This indicates that a moderate increase in the axial compression ratio can strengthen the cross-sectional integrity and initial stiffness, but an excessively high axial compression ratio will result in a sharp decline in the member’s strength reserve and accelerate structural failure. The other five types of specimens also show a similar law, and the specimens with larger cross-sectional dimensions have a relatively smaller decline in peak force, which suggests that increasing the cross-sectional dimensions can alleviate the adverse effects of high axial compression ratios. As can be observed from Figure 12, the force responses of all specimens exhibit significant fluctuation characteristics, with multiple peaks and valleys appearing in the curves. This phenomenon is mainly due to the structural properties of cold-formed thin-walled steel: first, the relatively thin wall thickness results in low local stiffness of the cross-section, making it prone to local buckling and indentation at the moment of impact, which causes a sudden drop in force; second, when stress waves propagate in thin-walled members, they reflect and superimpose when encountering screw connection points and cross-sectional corners, leading to high-frequency oscillations; in addition, the inconsistent deformation between individual limbs of the assembled cross-section and the relative slip at the screw connections further intensify the fluctuation in the force redistribution process. Such complex response characteristics indicate that the force transfer of thin-walled assembled columns under impact forces is a dynamic process involving multiple nonlinearities.

By comparing the response differences among the six types of specimens, it can be found that the specimen dimensional parameters have a regulating effect on the sensitivity to the axial compression ratio. The M-190 series specimens with larger cross-sectional dimensions exhibit better axial compression ratio tolerance than the M-140 series specimens. For instance, at an axial compression ratio of 0.6, the peak force reduction in the M-190-150-1.5 specimen is approximately 5% lower than that of the M-140-150-1.5 specimen. Meanwhile, he specimens with a steel thickness of 1.5 mm have a higher deformation control capacity than those with a 1.2 mm thickness, demonstrating that increasing the steel thickness can efficaciously delay the stiffness degradation process under high axial compression ratios. This size effect provides a basis for optimizing cross-sectional design, and the adverse effects of high axial compression ratios can be offset by reasonably configuring cross-sectional parameters. From the comprehensive analysis above, it can be concluded that the influence of the axial compression ratio on the impact resistance of CFS-LBC exhibits an obvious critical threshold effect. When the axial compression ratio is lower than 0.3, a moderate increase in the axial compression ratio can enhance the impact resistance by strengthening the cross-sectional constraint and friction effect; when the axial compression ratio exceeds 0.3, the P-Δ effect and the development of material plasticity dominate the response, leading to a sharp degradation of the specimens’ capacity to resist impact forces and their deformation capacity. Therefore, in practical engineering applications, it is advised that the axial compression ratio of such members be controlled below 0.3. If this requirement is difficult to meet, design strategies such as increasing the cross-sectional dimensions or steel thickness should be prioritized.

### 4.3. Effect of Impact Velocity

**Figure 13:**
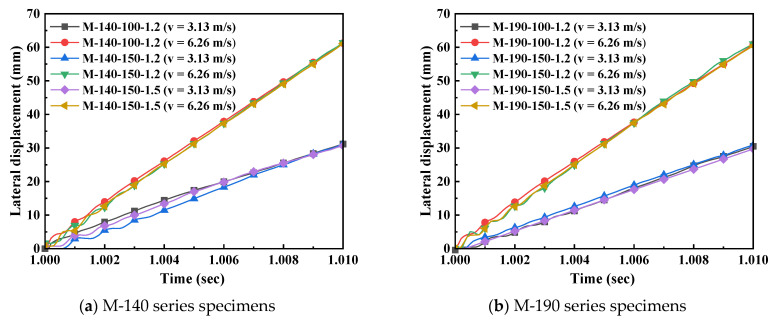
Displacement-time history curves (*n* = 0.3).

Impact velocity is a key parameter affecting the dynamic response of structural members, and it plays a decisive role in the impact resistance of CFS-LBC. By comparing and analyzing the dynamic responses of specimens under two impact velocities (3.13 m/s and 6.26 m/s), the influence of the velocity effect can be clearly observed. Figure 13 shows the displacement-time history curves of each specimen when the axial compression ratio is 0.3. It can be seen from the figure that, with an increase in impact velocity, the maximum lateral displacement of all specimens exhibits a significant increasing trend. Taking the M-140-100-1.2 specimen as an example, when the impact velocity increases from 3.13 m/s to 6.26 m/s, the maximum lateral displacement increases from 31.152 mm to 61.25 mm, with an increase rate of 96.6%. This phenomenon indicates that an increase in impact velocity causes the members to absorb more kinetic energy, thereby generating a larger deformation response.

**Figure 14:**
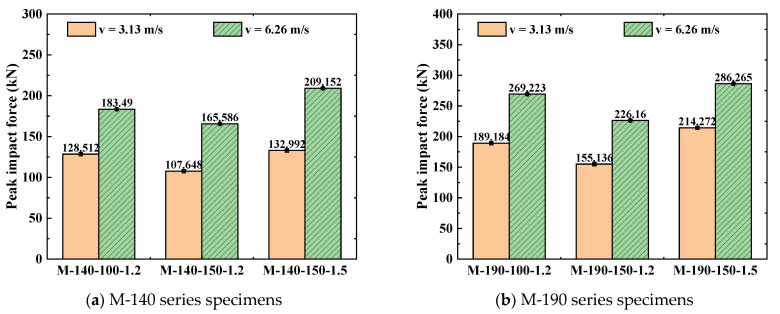
Peak lateral impact force of specimens with different velocities (*n* = 0.3).

It can be clearly observed from the bar chart of peak lateral impact force in Figure 14 that the increase in impact velocity significantly enhances the impact force response of the specimens. The peak force of all six types of specimens under the impact velocity of 6.26 m/s is significantly higher than that under the condition of 3.13 m/s. Taking the M-190-150-1.5 specimen as an example, its peak force is 214.272 kN at a velocity of 3.13 m/s; when the velocity increases to 6.26 m/s, the peak force rises to 286.265 kN, with an increase rate of 33.6%. This indicates that a higher impact velocity leads to a more intense force response. The reason for this is that the impact kinetic energy is proportional to the square of the velocity—doubling the velocity means the impact energy increases to four times, thus requiring a larger force to dissipate this energy.

By comparing the response differences among different series of specimens, it can be found that cross-sectional dimensions have a significant impact on velocity sensitivity. Compared with the M-140 series specimens, the M-190 series specimens exhibit a higher absolute value of peak force, but the force growth rate is relatively lower when the velocity increases. For example, the peak force of the M-190-100-1.2 specimen increases from 189.184 kN to 269.223 kN, with an increase rate of 42.3%; in contrast, the peak force of the M-140-100-1.2 specimen increases from 128.512 kN to 183.49 kN, with an increase rate of 42.8%. This indicates that although specimens with larger cross-sections have higher initial stiffness, the sensitivity of specimens with different dimensions to velocity changes is comparable, which suggests that the influence of impact velocity has a certain universality. Further analysis of the relationship between displacement response and force response reveals that an increase in impact velocity simultaneously amplifies both the displacement and force responses of the members, but there are differences in the growth amplitudes of the two. The growth amplitude of displacement is generally higher than that of force, which reflects the strain rate effect of steel materials. Under high-speed impact, the dynamic strength of the material increases, resulting in a rise in peak force; however, the significant increase in impact kinetic energy simultaneously results in a marked increase in deformation. This dual effect causes the members to experience both higher forces and greater deformations under high-speed impact, placing higher demands on the energy dissipation capacity of the members.

Based on the comprehensive analysis above, it can be concluded that impact velocity exerts a significant effect on the impact resistance of CFS-LBC. An increase in velocity not only amplifies the deformation response of the members but also increases the peak impact force. This velocity effect is universally present in specimens with different cross-sectional dimensions. In practical engineering applications, it is essential to fully consider the potential range of impact velocities and conduct targeted designs accordingly. For high-speed impact scenarios, it is advisable to use higher-strength materials or optimize cross-sectional design to boost the energy dissipation capacity of the members, thereby ensuring structural safety.

### 4.4. Effect of Specimen Dimensions

Specimen dimensional parameters have a remarkable impact on the impact resistance of CFS-LBC. By analyzing the response data of six specimens with different dimensions under the same axial compression ratio and impact velocity, the influence laws of parameters such as cross-sectional size, steel thickness, and screw spacing can be systematically studied. It can be clearly observed from Table 5 that differences in specimen dimensions result in significant variations in the mechanical properties of the specimens.

From the comparison of peak lateral impact forces, it is evident that an increase in cross-sectional dimensions significantly improves the specimens’ capacity to resist impact forces. The peak forces of the M-190 series specimens are generally higher than those of the M-140 series specimens. Among them, the peak force of the M-190-150-1.5 specimen reaches 214.272 kN, while that of the M-140-150-1.5 specimen is only 132.992 kN, representing an increase rate of 61.1%. This phenomenon indicates that increasing the cross-sectional dimensions can effectively enhance the cross-section’s flexural stiffness and impact resistance. This is because the increase in cross-sectional dimensions significantly increases the moment of inertia of the cross-section, thereby improving the overall stiffness. Changes in the steel thickness parameter also have a remarkable impact on the specimen performance. By comparing specimens with the same cross-sectional height and screw spacing, it can be found that increasing the steel thickness from 1.2 mm to 1.5 mm can significantly increase the peak force. Taking the M-140 series as an example, the peak force of the M-140-150-1.5 specimen is 23.5% higher than that of the M-140-150-1.2 specimen. This indicates that increasing the steel thickness can effectively improve the local stability of the cross-section, reduce local buckling deformation under impact forces, and thereby enhance the overall force transfer efficiency. The influence of the screw spacing parameter also cannot be ignored. It can be observed from the data in Table 5 that, under the same cross-sectional height and steel thickness conditions, reducing the screw spacing increases the peak force of the specimens. As an illustration, the peak force of the M-190-100-1.2 specimen is 189.184 kN, while that of the M-190-150-1.2 specimen is 155.136 kN, a decrease of 18.0%. This shows that a smaller screw spacing can enhance the integrity of the assembled cross-section and improve the cooperative working capacity between the two assembled components, thereby resisting impact forces more effectively.

From the perspective of displacement response, changes in dimensional parameters also affect the deformation characteristics of the specimens. The maximum lateral displacement of the M-190 series specimens is generally smaller than that of the M-140 series specimens, indicating that increasing the cross-sectional dimensions can effectively control deformation. Meanwhile, an increase in steel thickness also shows a trend of reducing deformation: the maximum lateral displacement of the M-140-150-1.5 specimen is 30.636 mm, which is smaller than the 31.292 mm of the M-140-150-1.2 specimen. This suggests that by optimizing dimensional parameters, structural deformation can be controlled while improving the peak force. The data on axial displacement in Table 5 further reveals the influence mechanism of dimensional parameters. The maximum axial displacement of the M-190 series specimens is significantly smaller than that of the M-140 series specimens. For instance, the axial displacement of the M-190-100-1.2 specimen is only 0.522 mm, while that of the M-140-100-1.2 specimen reaches 1.3 mm. This indicates that specimens with larger cross-sections possess better axial stiffness and can more effectively resist compressive deformation caused by impact, which is consistent with their higher peak force performance.

Based on the comprehensive analysis above, it can be concluded that the dimensional parameters of specimens exert a systematic influence on their impact resistance. Increasing the cross-sectional dimensions, increasing the steel thickness, and reducing the screw spacing can all effectively enhance the peak impact force of the specimens and control the development of deformation. In practical engineering applications, it is advisable to prioritize the use of larger cross-sectional heights, appropriately increase the steel thickness, and adopt smaller screw spacing to optimize the impact resistance of CFS-LBC. The optimized design of these dimensional parameters provides an effective technical approach for improving the impact resistance of such structures.

## 5. Conclusions

In this study, an FEM for CFS-LBC under lateral impact forces was developed. The modeling methodology was based on techniques previously validated against axial compression tests [11]. The validated model was then used for systematic parametric analysis to investigate the effects of axial compression ratio, impact velocity, and specimen dimensional parameters on the dynamic response and failure characteristics of the members under transverse impact forces. The main conclusions are as follows:

(1) The impact of the axial compression ratio on the impact resistance of members exhibited a significant nonlinear characteristic. As the axial compression ratio increased from 0.1 to 0.3, the peak impact force increased by an average of 15.8% owing to the restraining effect of axial pressure on initial defects; nevertheless, when the axial compression ratio further increased to 0.6, the peak impact force decreased significantly by 38.6% due to the intensified P-Δ effect and exhaustion of strength reserve. This finding indicated that there existed an optimal axial compression ratio of 0.3, which ought to be regarded as an important design control value in practical engineering.

(2) Impact velocity exerted a decisive impact on the dynamic response of the members. The research results showed that as the impact velocity increased from 3.13 m/s to 6.26 m/s, the peak impact force of the specimens rose by an average of 38.2%, and the maximum lateral displacement increased by 96.5%. This indicated that under high-speed impact, the deformation response of the members was more sensitive than the force response, and the growth rate of displacement reached 2.5 times that of the force.

(3) Specimen dimensional parameters significantly affected the impact resistance of the members. Increasing the cross-sectional dimension from 90 mm to 140 mm increased the peak impact force by 61.1%; increasing the steel thickness from 1.2 mm to 1.5 mm increased the force by 23.5%; and reducing the screw spacing from 150 mm to 100 mm enhanced the force by 18.0%. These quantitative relationships suggested that the impact resistance of the members could be significantly improved through reasonable parameter configuration.

(4) Extra care must be taken regarding the coupling effect between the axial compression ratio and impact velocity. Under the combined action of a relatively high axial compression ratio (0.6) and high-speed impact (6.26 m/s), the axial displacement of the members could increase by 356%, which indicated that the coupling effect would drastically amplify the dynamic response of the structure. Therefore, in actual engineering projects, it is essential to avoid the simultaneous occurrence of a high axial compression ratio and high-speed impact.

(5) The impact resistance of the members could be significantly enhanced by optimizing the specimen dimensional parameters. Increasing the cross-sectional dimensions and steel thickness could boost the stiffness and strength of the members, while reducing the screw spacing could improve the integrity of the cross-section. When these measures were applied together, the impact resistance of the members could be comprehensively improved, which provided clear design guidance for engineering practice.

In the following work, we will further investigate the mechanical properties of the members under different impact positions, boundary conditions, and complex force cases. Meanwhile, more experimental studies can be carried out to verify the reliability of the numerical simulation results, and the refined modeling of material strain rate effects and failure criteria should be considered. In addition, establishing simplified design calculation formulas based on a large number of parametric analysis results will be an important direction to promote the engineering application of this research achievement.

## Figures and Tables

**Table 1:** Dimensions of built-up columns.

Specimen Number	Screw Spacing (mm)	*t* (mm)	*D* (mm)	*h*_1_ (mm)	*h* (mm)	*l* (mm)
M-140-100-1.2	100	1.2	140	50	90	1500
M-140-150-1.2	150	1.2				
M-140-150-1.5	150	1.5				
M-140-300-1.2	300	1.2				
M-190-100-1.2	100	1.2	190	50	140	
M-190-150-1.2	150	1.2				
M-190-150-1.5	150	1.5				

**Table 2:** Comparison of bearing capacities between tests and finite element analyses [11].

Specimen Number	*P*_t_/kN	*P*_A_/kN	*P*_A_/*P*_t_
M-140-100-1.2	108.40	115.95	1.070
M-140-150-1.2	108.80	115.60	1.063
M-140-150-1.5	183.45	184.35	1.005
M-140-300-1.2	111.75	116.49	1.042

Note: *P*_A_ and *P*_t_ represent the ultimate capacity from finite element analysis and the ultimate capacity from tests, respectively.

**Table 3:** Stress–strain relationship.

**T1.2**	ε (×10^−2^)	0	0.17	0.20	2.01	3.36	7.61	12.32	23.36	24.05
	σ (MPa)	0	344.28	395.1	397.82	439.37	500.78	520.75	515.21	470.07
**T1.5**	ε (×10^−2^)	0	0.16	0.20	1.85	3.34	8.33	17.06	21.63	22.32
	σ (MPa)	0	321.53	395.0	397.82	437.23	500.26	521.35	515.96	472.11

**Table 4:** Axial loads applied corresponding to different axial compression ratios of specimens.

Specimen Number	n	Axial Load (kN)	n	Axial Load (kN)	n	Axial Load (kN)
M-140-100-1.2	0.1	10.8	0.3	32.4	0.6	64.8
M-140-150-1.2		10.9		32.7		65.4
M-140-150-1.5		18.3		54.9		109.8
M-190-100-1.2		11.4		34.2		68.4
M-190-150-1.2		11.7		35.1		70.2
M-190-150-1.5		19.8		59.4		118.8

**Table 5:** Effect of specimen dimensional parameters on their dynamic responses (*n* = 0.3, *v* = 6.26 m/s).

Specimen Number	Maximum Lateral Displacement (mm)	Maximum Axial Displacement (mm)	Peak Lateral Impact Force (kN)
M-140-100-1.2	31.152	1.3	128.512
M-140-150-1.2	31.292	1.218	107.648
M-140-150-1.5	30.636	1.298	132.992
M-190-100-1.2	30.487	0.522	189.184
M-190-150-1.2	30.868	0.718	155.136
M-190-150-1.5	29.689	0.68	214.272

## Data Availability

The original contributions presented in this study are included in the article. Further inquiries can be directed to the corresponding author.
